# Inhibition of TRPV1 Channel Activity in Human CD4^+^ T Cells by Nanodiamond and Nanoplatinum Liquid, DPV576

**DOI:** 10.3390/nano8100770

**Published:** 2018-09-29

**Authors:** Mamdooh H. Ghoneum, James K. Gimzewski, Aya Ghoneum, Hideki Katano, Clarissa Nila Paw U, Anshu Agrawal

**Affiliations:** 1 Department of Surgery, Charles Drew University of Medicine and Science, Los Angeles, CA 90059, USA; 2 Department of Chemistry and Biochemistry, UCLA, 607 Charles E. Young Drive East, Los Angeles, CA 90095, USA; gimzewski@cnsi.ucla.edu (J.K.G.); ayaghoneum@ucla.edu (A.G.); 3 California NanoSystems Institute (CNSI) at UCLA, 570 Westwood Plaza, Los Angeles, CA 90095, USA; 4 Department of Regenerative Medicine, Tokai University School of Medicine, 143 Shimokasuya, Isehara, Kanagawa 259-1143, Japan; katano@venex-j.co.jp; 5 Division of Basic and Clinical Immunology, Department of Medicine, University of California, Irvine, CA 92697, USA; cpawu@uci.edu (C.N.P.U.); aagrawal@uci.edu (A.A.)

**Keywords:** nanodiamond, CD4^+^ T cells, TRPV1, capsaicin

## Abstract

**Background:** Transient receptor potential vanilloid (TRPV) channels act as sensors of pain, temperature, and other external stimuli. We have recently shown that DPV576, an aqueous mixture of nanodiamond (ND) and nanoplatinum (NP), can modulate the activity of TRPV on human primary keratinocytes, suggesting their potential as a possible pain modulator. Here, we sought to examine the effect of DPV576 in modulating the functions of human CD4^+^ T lymphocytes and whether the modulation is mediated via TRPV channels. **Materials and methods:** Human primary CD4^+^ T cells were activated with anti CD3/CD28 with and without DPV576 at 1:10 and 1:100 dilutions for 24 h in vitro. TRPV receptor expression (TRPV1 and TRPV4) on CD4^+^ T cells were examined by flow cytometry. The capacity of DPV576 to modulate the activity of TRPV1 agonist capsaicin in CD4^+^ T cells was also determined. Activation of CD4^+^ T cells was determined by production of cytokines TNF-α, IFN-γ, and IL-10 using specific ELISA kits. **Results:** DPV576 treatment of CD4^+^ T cells that were activated with anti CD3/CD28 resulted in decreased expression of the TRPV1 channel, but had no effect on TRPV4. This was accompanied by decreased secretion of IFN-γ and reduced expression of TRPV1 in capsaicin activated CD4^+^ T cells. In addition, DPV576 inhibited the capsaicin, induced the production of IFN-γ, and enhanced the secretion of IL-10. **Conclusion:** We conclude that short term exposure to DPV576 inhibits the activity of TRPV1 channels in CD4^+^ T lymphocytes, which may suggest its possible beneficial use for pain management.

## 1. Introduction

Tissue damage, due to noxious physical, chemical, or mechanical stimuli, constitutes a major challenge to homeostasis, which disrupts tissue integrity and triggers a coordinated and diverse response mediated by effector cells, such as immune cells, peripheral neurons, fibroblasts, and endothelial cells. A hallmark feature of such damage is the release of inflammatory mediators that activate receptors, leading to altered cellular activities. Many of these mediators bind to receptors expressed on afferent terminals, leading to dramatic changes in neuronal excitability [1].

The family of transient receptor potential (TRP) is an evolutionarily conserved group of ligand-gated ion channels that respond to a variety of stimuli, including variations in temperature, pH, osmolarity, pro-inflammatory agents, and multiple endogenous or exogenous stress mediators [2]. Because of their property to respond to the thermal and chemical stimuli, TRP channels can activate sensory neurons to induce pain [3]. Our work and that of others showed that members of the TRP family are expressed in a wide variety of different cell types, such as neurons, skin, mesenchymal stem cells, vascular cells, fibroblasts, and immune cells [4,5,6].

Vanilloid (TRPV) is one of the six families of TRP. The TRPV family can be divided into two subfamilies: the first subfamily comprises TRPV1-4, acts as a non-store operated Ca^2+^ channel [7], and is known to participate in thermo-sensation [2]; the other subfamily comprises TRPV5/6, is exclusively permeable to Ca^2+^, and is viewed as the gatekeeper of epithelial calcium transport [4,8,9]. TRPV channels are expressed on immune cells, such as in human blood lymphocytes [10], in normal human T lymphocytes [11], and in CD4^+^ T cells [7,12].

Inflammatory cytokines produced by immune cells act on the neurons to induce pain. These cytokines are also considered major fatigue and stress inducers [7]. The production of inflammatory cytokines in immune cells can be modulated by TRPV channels. In this regard, it has been shown that genetic deletion or pharmacological inhibition of TRPV1 in CD4^+^ T cells substantially reduced colitis severity in animal models of human inflammatory bowel disease [7]. These data suggest that targeting the TRPV channel could represent a novel strategy to inhibit pro-inflammatory CD4^+^ T cell responses in related human diseases. In addition to inflammation, compounds aimed at decreasing the expression of TRPV channels are also emerging as major painkillers. An example is capsaicin I-RTX (a specific TRPV1 antagonist) [13]. We have recently shown that a dispersed aqueous mixture of nanodiamond (ND) and nanoplatinum (NP) (DPV576) can down-regulate the expression of TRPV4 on human primary keratinocytes, suggesting their potential as a possible pain modulator [14]. The current study was carried out to further expand the potential role of DPV576 on inflammatory responses induced by TRPV1 activation on CD4^+^ T lymphocytes. These results have the potential to facilitate the design of future combinations of ND and NP therapeutics aimed at modulating local inflammation and pain. To our knowledge, this study is the first investigation into these potential therapeutic properties of nanoparticles.

## 2. Methods

### 2.1. Blood Donors

Peripheral blood samples were obtained from healthy volunteers under an Institutional Review Board (IRB) approved protocol.

### 2.2. DPV576 Liquid

An aqueous mixture of nanodiamond (ND) and nanoplatinum (NP) mixture solution known as DPV576 was used [15]. This mixture is composed of Platinum (0.03 μg/mL) and Diamond (2 μg/mL). Other details regarding particle size, shape, and composition of the ND and NP mixture have been described earlier [15]. DPV576 was supplied by Venex Co, Ltd., Kanagawa, Japan.

### 2.3. CD4^+^ T Cell Purification

CD4^+^ T cells were purified from the PBMCs by negative selection using a CD4^+^ T cell enrichment kit (Stemcell Technologies, Vancouver, BC, Canada), with purity above 90%.

### 2.4. Effect of DPV576 on T Cells Activation

Purified CD4^+^ T cells were stimulated with anti-CD3 and anti-CD28 beads (Stemcell Technologies) in RPMI medium containing 10% fetal bovine serum (FBS). DPV576 was added simultaneously at concentrations of 1:10 and 1:100 for 24 h. The expression of TRPV1 and TRPV4 on the cells was determined using flow cytometry. Briefly, cells were collected and centrifuged, re-suspended in PBS (phosphate buffered saline) 2% FBS, and incubated with specific antibodies for TRPV1-AL647 (bs-1931R-A647) or TRPV4-Alexa 488 (bs-6425R-A488) (Bioss Inc., Woburn, MA, USA) at a dilution of 1:100 for 1 h. Subsequently, the cells were washed and a minimum of 10,000 cells were acquired on FACS Calibur (Becton Dickinson, San Jose, CA, USA). Isotype antibodies (bs-0295P-A647, bs-0295P-A488, Bioss) were used as controls. Analysis of the expression of TRPVs was performed by FlowJo (FlowJo, LLC, Ashland, OR, USA). Supernatants collected were assayed for cytokines IFN-γ, TNF-α and IL-10 using specific ELISA kits (BD Biosciences, Franklin Lakes, NJ, USA).

### 2.5. Stimulation with Anti-CD3 and Anti-CD28 Beads in the Presence and Absence of TRPV1 Inhibitor SB366791 and DPV576

Purified CD4^+^ T cells were stimulated with anti-CD3 and anti-CD28 beads (Stemcell Technologies) plus DPV576 concentrations of 1:10 and 1:100 for 24 h in the presence or absence of the TRPV1 inhibitor, SB366791, at a concentration of 10 µMol/mL (Tocris, UK). Supernatants collected were assayed for cytokines IFN-γ, TNF-α, and IL-10 using specific ELISA kits (BD Biosciences, Franklin Lakes, NJ, USA).

### 2.6. Stimulation with Anti-CD3 and Anti-CD28 Beads in the Presence and Absence of Capsaicin and DPV576

Purified CD4^+^ T cells were stimulated with anti-CD3 and anti-CD28 beads (Stemcell Technologies) plus DPV576 concentrations of 1:10 and 1:100 for 24 h in the presence or absence of capsaicin 1 µMol/mL (Sigma, St. Louis, MO, USA). Supernatants collected were assayed for cytokines IFN-γ, TNF-α, and IL-10 using specific ELISA kits (BD Biosciences, Franklin Lakes, NJ, USA).

### 2.7. Statistical Analysis

Statistical analysis for experiments was performed using GraphPad Prism (GraphPad Inc., San Diego, CA, USA). T-test was used for analysis of 2 groups. One-way ANOVA followed by Tukey’s test was used with 3 or more groups and was used for analysis. A *p*-value of <0.05 was considered statistically significant.

## 3. Results

### 3.1. DPV576 Down Modulates the Expression of TRPV1 on Activated CD4^+^ T Cells

**Figure 1:**
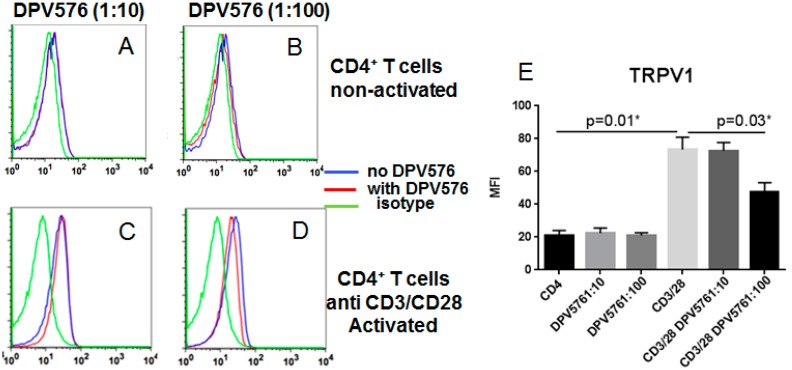
(**A**–**E**) DPV576 down modulates the expression of TRPV1 on activated CD4^+^ T cells. Non-activated and anti-CD3/CD28-activated CD4^+^ T lymphocytes were exposed to DPV576 for 24h. The cells were stained for transient receptor potential vanilloid (TRPV) channels. Expression of TRPV1 on non-activated T cells (**A**,**B**). Anti-CD3/CD28 activated T cells with DPV576 (**C**,**D**). Data is representative of three such experiments. Blue: Without DPV576; Red: DPV576-exposed lymphocytes; Green: Isotype. Bar graph depicts the mean ± S.E. of MFI of the groups (**E**).

We determined the expression of TRPV1 channels on CD4^+^ T cells with and without DPV576 using flow cytometry. First, we examined the effect of DPV576 on expression of TRPV1 on non-activated CD4^+^ T cells. As shown in Figure 1A–E, exposure of CD4^+^ T cells to DPV576 in the absence of T cell activation did not have a significant effect on the expression of TRPV1 on CD4^+^ T cells.

Second, we examined the effect of DPV576 on expression of TRPV1 on CD4^+^ T cells activated with anti-CD3/CD28. Activation of T cells resulted in the upregulation of TRPV1 expression. However, when T cells were activated with anti-CD3 and anti-CD28 beads in the presence of DPV576, we observed downregulation of TRPV1 expression over control cells (Figure 1D,E) at an ND concentration of 1:100. These data suggest that DPV576 down modulates the expression of TRPV1 on activated CD4^+^ T cells.

### 3.2. DPV576 Does Not Modulate the Expression of TRPV4 on CD4^+^ T Cells

**Figure 2:**
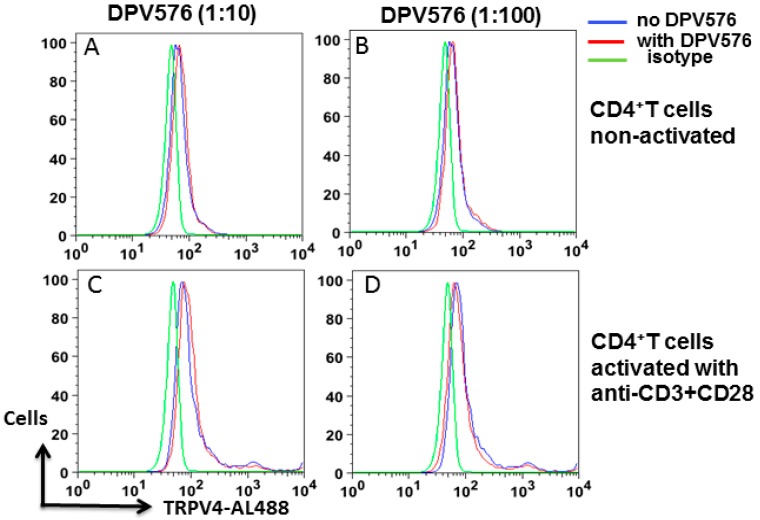
(**A**–**D**) DPV576 does not modulate the expression of TRPV4 on CD4^+^ T cells. Inactivated and anti-CD3/CD28 activated CD4^+^ T lymphocytes were exposed to DPV576 for 24 h. The cells were stained for TRPV channels. Expression of TRPV4 on non-activated T cells (**A**,**B**). Anti-CD3/CD28 activated T cells (**C**,**D**). Data is representative of three experiments. Blue: Without DPV576; Red: DPV576 exposed lymphocytes; Green: Isotype.

Next, we examined whether DPV576 also affected the expression of TRPV4 on CD4^+^ T cells. As shown in Figure 2A–D, exposure of CD4^+^ T cells to DPV576 in the absence of T cell activation did not have a significant effect on the expression of TRPV4 on CD4^+^ T cells (Figure 2A,B). Similarly, there was no significant effect on the expression of TRPV4 on anti-CD3/CD28 activated CD4^+^ T cells (Figure 2C,D). These data suggest that DPV576 has no effect on the expression of TRPV4 on CD4^+^ T cells.

### 3.3. DPV576 Modulates Cytokine Secretion from Activated CD4^+^ T Cells

**Figure 3:**
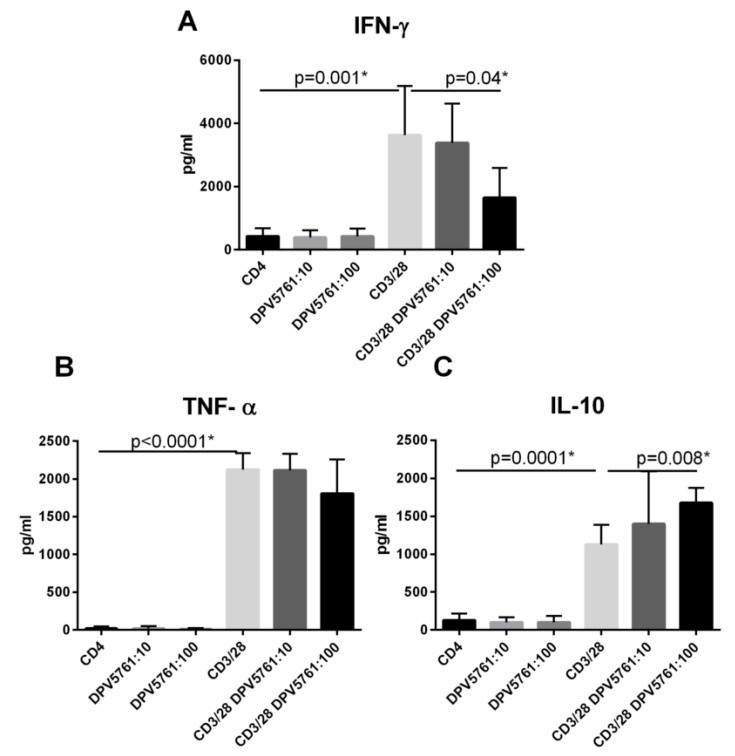
(**A**–**C**) DPV576 modulates cytokine secretion from CD4^+^ T cells. Non-activated and anti CD3/CD28 activated CD4^+^ T lymphocytes were cultured in the presence of DPV576 for 24 h. Secretion of cytokines was determined by ELISA. Bar graph depicts the level of: (**A**) IFN-γ, (**B**) TNF-α, and (**C**) IL-10. Data is mean ± S.E. of three experiments.

Cytokine secretion by T cells is a measure of their inflammatory activity; therefore, we examined the effect of DPV576 on cytokine secretion by CD4^+^ T cells. Data in Figure 3A–C shows that exposure of inactivated CD4^+^ T lymphocytes to DPV576 shows undetectable levels of the cytokines IFN-γ, TNF-α, and IL-10. However, activation of CD4^+^ T cells with anti-CD3 and anti-CD28 beads followed by exposure to DPV576 resulted in decreased secretion of IFN-γ (Figure 3A) at a concentration of 1:100. Reduction in TNF-α was not significant, but IL-10 was significantly enhanced over controls in the activated CD4^+^ T cells (Figure 3B,C). These data suggest that DPV576 inhibits IFN-γ and induces IL-10 secretion in CD4^+^ T cells.

### 3.4. TRPV1 Antagonist, SB366791, Inhibits the Effect of DPV576 on Cytokine Secretion by CD4^+^ T Cells

**Figure 4:**
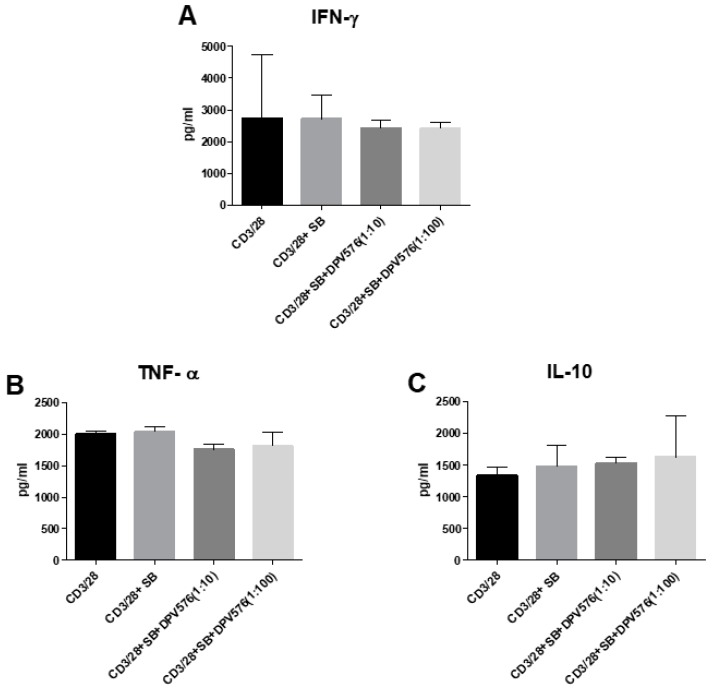
(**A**–**C**) TRPV1 antagonist, SB366791 (SB), inhibits the effect of DPV576 on cytokine secretion by CD4^+^ T cells. SB + anti CD3/CD28 activated CD4^+^ T lymphocytes were exposed to DPV576 for 24 h. Secretion of cytokines was determined by ELISA. Bar graph depicts the level of: (**A**) IFN-γ, (**B**) TNF-α, and (**C**) IL-10. Data is mean ± S.E. of three experiments.

DPV576 reduced the expression of TRPV1 (Figure 1D–E) and also suppressed IFN-γ secretion from CD4^+^ T cells (Figure 3A–C). However, this does not confirm that inhibition of cytokine secretion by DPV576 is mediated via down regulation of TRPV1. Therefore, to confirm this, anti-CD3/CD28 activated T cells were cultured together with selective TRPV1 antagonist, SB366791 (SB), in the presence or absence of DPV576, and cytokine secretion was determined after 24 h. As shown in Figure 4A, incubation with SB prevented the DPV576 mediated inhibition of IFN-γ. The increase in IL-10 was also abrogated (Figure 4C). These data confirm that DPV576 acts via TRPV1 in CD4^+^ T cells.

### 3.5. DPV576 Synergizes with Capsaicin to Downregulate the Expression of TRPV1 on CD4^+^ T Cells

**Figure 5:**
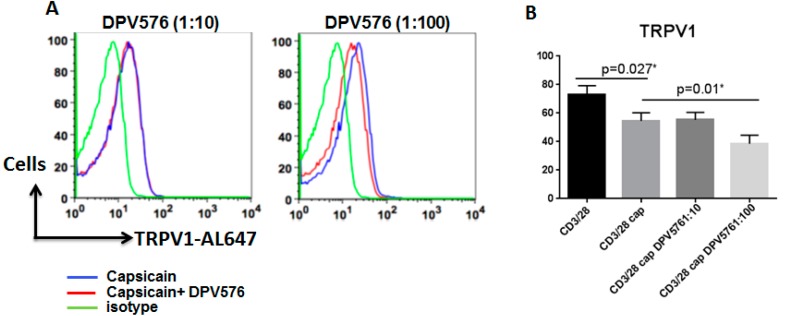
DPV576 downregulated the expression of TRPV1 in capsaicin activated in CD4^+^ T cells. Anti-CD3/CD28 + capsaicin activated CD4^+^ T lymphocytes in the presence of DPV576 for 24 h. The cells were stained for TRPV1. (**A**) Data is representative of three experiments. Blue: capsaicin; Red: capsaicin + DPV576 exposed lymphocytes; Green: anti-CD3/CD28. (**B**) Bar graph depicts the mean ± S.E. of the same.

To investigate the effect of DPV576 on the TRPV1 agonist, capsaicin-induced TRPV1 activity, we determined the expression of TRPV1 on anti-CD3 and anti-CD28 activated CD4^+^ T cells stimulated with capsaicin in the presence or absence of DPV576. Results of flow cytometry suggest that capsaicin downregulated the expression of TRPV1 on CD4^+^ T cells, which is further downregulated in the presence of DPV576 (Figure 5A,B). Thus, DPV576 synergizes with capsaicin to desensitize TRPV1.

### 3.6. DPV576 Modulates Cytokine Secretion of TRPV1 Agonist, Capsaicin, in CD4^+^ T Cells

**Figure 6:**
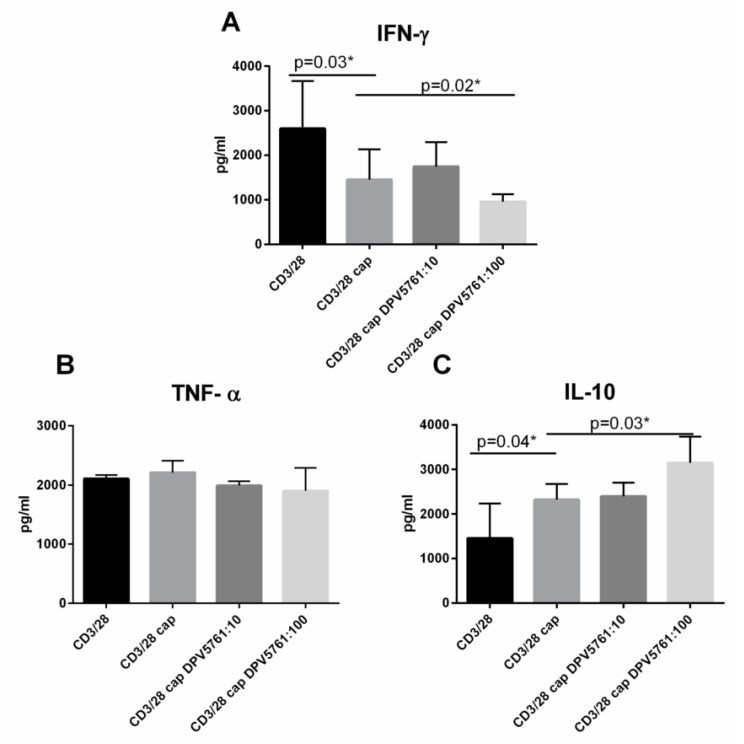
(**A**–**C**) DPV576 modulated cytokine secretion of TRPV1 agonist, capsaicin, in CD4^+^ T cells. Capsaicin + anti CD3/CD28 activated CD4^+^ T lymphocytes were exposed to DPV576 for 24 h. Secretion of cytokines was determined by ELISA. Bar graph depicts the level of: (**A**) IFN-γ, (**B**) TNF-α, and (**C**) IL-10. Data is mean ± S.E. of three experiments.

We observed a decrease in TRPV1 expression by DPV576 in the capsaicin-induced downregulation of TRPV1 in CD4^+^ T cells (Figure 5), which suggests that DPV576 enhances the activity of capsaicin. To investigate this possibility, anti-CD3 and anti-CD28 activated CD4^+^ T cells were stimulated with capsaicin in the presence or absence of DPV576. Cytokine secretion after 24 h was determined by ELISA. Results in Figure 6 indicate that stimulation of CD4^+^ T cells with capsaicin alone led to a slight decrease in the secretion of IFN-γ and an increase of IL-10. The addition of DPV576 to capsaicin-primed CD4^+^ T cells caused the following: (1) inhibition of IFN-γ secretion; (2) increased production of IL-10; and (3) no effect on TNF-α production. These results further confirm that DPV576 synergizes with capsaicin to modulate the activity of TRPV1 channels in CD4^+^ T cells.

## 4. Discussion

Several studies have demonstrated the role of TRPV1 receptors in T cell activation and functions [11,16]. Here, we examined whether an aqueous mixture of ND and NP, DPV576, can modulate the activity of these receptors on CD4^+^ T cells. We observed decreased expression of TRPV1 over control cells when T cells were activated with anti-CD3 and anti-CD28 beads. Moreover, DPV576 inhibits the expression of TRPV1 in capsaicin activated CD4^+^ T cells. DPV576-induced TRPV1 expression was associated with significant inhibition in the secretion of IFN-γ and increase in IL-10. These results show that DPV576 can modulate TRPV1 activity in CD4^+^ T cells and that it also synergizes with the TRPV1 agonist, capsaicin, to further desensitize TRPV1.

Data of the current study reveal the expression of TRPV1 on CD4^+^ T cells. Several studies demonstrated TRPV1 mRNA and protein expression in primary mouse and human T cells [7,17,18,19,20] and in mouse and rat thymocytes [21,22]. Earlier studies reveal that T cell activation and release of specific inflammatory cytokines, which may be associated with immune-related diseases, are attributed to several TRP channels that are expressed in T cells [6,23]. Special emphasis was focused on the role of TRPV1 that has been examined in vivo in models of T cell-mediated colitis via its pro-inflammatory properties and regulation of cytokine production [7,24,25]. Data of the current study showed that short term exposure to DPV576 modulates the activity of TRPV1 channels in CD4^+^ T lymphocytes, which is associated with decreased production of IFN-γ at both 1:10 and 1:100 dilutions of DPV576.

It has been generally accepted that TRP channel inhibitors have the ability to block receptors responsible for pain generation. For example, the discovery of archetypal thermoTRP, the vanilloid (capsaicin) receptor TRPV1, nearly two decades ago has piqued considerable interest in the scientific community [6,26,27]. TRPV1 became the focus of research as pain perception. Despite extensive research, the underlying mechanisms of pain are still not fully understood. In recent years, increasing evidence indicates a pivotal role of the immune system in pain [28,29]. The majority of previously published data links pain syndromes with higher levels of pro-inflammatory cytokines. Emerging evidence also suggests a role of T-lymphocytes in chronic neuropathic pain [30]. A TH1/TH2 imbalance has already been shown in patients with complex regional pain syndrome and chronic pelvic pain [31,32]. TH17 has been linked to increased pain sensitivity and destructive effects promoting persistent pain [33], while Tregs were found to be mainly involved in the endogenous recovery [34]. Our data showed the ability of DPV576 to down modulate TRPV1 and IFN-γ on CD4^+^ T cells, suggesting that these cells play a central role in pain management and also illustrates a possible beneficial effect of DPV576 in modulating pain and fatigue. Enhanced production of the anti-inflammatory cytokine, IL-10 (Figure 3C), will further aid in reducing pain by desensitizing TRPV1. Furthermore, data showed that treatment with DPV576 inhibits the expression of TRPV1 in capsaicin-activated CD4^+^ T cells, which suggests that it can have an additive effect and can enhance the desensitizing activity of capsaicin [35].

Our current and earlier studies examined the effects of DPV576 on two different types of cells, keratinocytes versus CD4^+^ T cells. In the previous study on keratinocytes [14], we found that the expression of TRPV1 did not change significantly while the expression of TRPV4 decreased upon treatment with DPV576. On the other hand, for the current study with CD4^+^ T cells, we observed the reverse: the expression of TRPV1 decreased significantly while the expression of TRPV4 did not change significantly. Taken together, these results indicate a cell-dependent differential response between TRPV1 and TRPV4 upon treatment with DPV576 that may be related to the differences in environments in which each channel is stimulated [36,37]. Another study has shown that the response of TRPV1 can be different even within the same tissue, for example, in large vs. small arteries [38]. In this study, the authors state, “it appears that the TRPV1 is not uniformly expressed in the vascular tissue, with TRPV1 only expressed in a subset of blood vessels in some tissues (in particular, mesenteric arteries and skin)” [38].

Proinflammatory cytokines can cause changes in behavior, including symptoms of fatigue, lethargy, muscle aches, cognitive dysfunction, and depressed mood [39,40]. Interestingly, results of a recent and robustly designed study by Raison et al. showed that all levels of fatigue are associated with increased inflammation, as indexed by elevated plasma C-reactive protein levels and white blood cell count, even after adjusting for depressive status [39]. This study further supports the notion that the symptom of fatigue, rather than a diagnosis of chronic fatigue syndrome itself, may be what is clinically associated with inflammation. Decreased secretion of IFN-γ by CD4^+^ T cells in the presence of DPV576 may thus be beneficial in reducing fatigue and muscle aches. This could be one of the potential uses of clothes incorporating nanodiamond (ND) and nanoplatinum (NP).

In summary, DPV576 inhibits the activity of TRPV1 in CD4^+^ T lymphocytes. It also enhances the activity of the TRPV1 agonist, capsaicin. These results indicate the therapeutic potential of using DPV576 to block the activity of TRPV1, which is the receptor for pain and other noxious stimuli.
